# Simultaneous use of oxalate-degrading bacteria and herbal extract to reduce the urinary oxalate in a rat model: A new strategy

**DOI:** 10.1590/S1677-5538.IBJU.2019.0167

**Published:** 2019-12-17

**Authors:** Rouhi Afkari, Mohammad Mehdi Feizabadi, Alireza Ansari-Moghadam, Tahereh Safari, Mohammad Bokaeian

**Affiliations:** 1 Infectious Diseases and Tropical Medicine Research Center, Resistant Tuberculosis Institute, Zahedan University of Medical Sciences, Zahedan, Iran; 2 Tehran University of Medical Sciences (TUMS), Tehran, Iran; 3 School of Health, Health Promotion Research Center, Zahedan University of Medical Sciences, Zahedan, Iran; 4 Zahedan University of Medical Sciences, Zahedan, Iran

**Keywords:** Probiotics, Jeevaneeya Rasayana [Supplementary Concept], cationic protein, urate-calcium oxalate stone, human [Supplementary Concept]

## Abstract

**Objective:**

Urinary stones with oxalate composition can cause kidney failure. Recent findings evidenced that probiotics are effective in reducing oxalate absorption in these subjects based on their high colonic absorption levels at baseline. The purpose of this study was to evaluate the effect of the simultaneous use of oxalate-degrading bacteria, Urtica dioica and T. terrestris extract in reducing urinary oxalate.

**Materials and Methods:**

Anti-urolithiatic activity of Urtica dioica and T. terrestris extract and probiotic by using ethylene glycol induced rat model. In this study, 4 strains of Lactobacillus and 2 strains of Bifidobacterium and also 2 strains of L. paracasei (that showed high power in oxalate degrading in culture media) were used. Male Wistar rats were divided into four groups (n=6). The rats of group-I received normal diet (positive control group) and groups-II (negative control group), III, IV rats received diet containing ethylene glycol (3%) for 30 days. Groups III rats received Urtica dioica and T. terrestris extract. Groups IV rats received extracts + probiotic for 30 days.

**Findings:**

The results show that the use of herbal extracts (Urtica dioica and T. terrestris) reduced the level of urinary oxalate and other parameters of urine and serum. Also, the accumulation of calcium oxalate crystals in the kidney tissue was significantly reduced.

**Conclusion:**

Considering that the formation of calcium oxalate crystals can cause inflammation and tissue damage in the kidney, the use of herbal extracts with oxalate degrading bacteria can be a new therapeutic approach to preventing the formation of kidney stones.

## INTRODUCTION

Today, kidney stones represent an important health problem in many countries. Genetic factors, metabolic disturbances (excess oxalate synthesis), food and environmental factors are among the most important causes of kidney stones, with 60-80% of human kidney stones induced by calcium oxalate (1, 2). In fact, hyperoxaluria is one of the major risk factors of calcium oxalate stone formation due to urinary calcium oxalate supersaturation. Oxalates in the human body increase both in endogenous (during the synthesis of ascorbic acid) and exogenous(oxalate-rich foods) (2, 3). There is a direct correlation between oxalate dietary intake and the formation of calcium oxalate stones. It is believed that the consumption of oxalate foods only increase 50-60% urinary oxalate (4). Generally, increasing urinary oxalate, causes the destruction of kidney tissues, acute renal failure and the formation of oxalate crystals in the urethra (3–5). Based on several studies, medical herbs contain active substances with a therapeutic effect on the kidney and urinary tract system (6). Indeed, in Iranian traditional and ethnobotanical medicine, medical herbs are used to treat kidney diseases and disorders. The traditional medicine has now gained recognition all over the world with several indigenous drugs forming an indispensable part of health care (5). Tribulus terrestris is an annual plant in the caltrop family (Zygophyllaceae) which is widely distributed worldwide. It is adapted to grow in dry climate locations in which few other plants can survive, and is an invasive species in Iran and India (7). As with many weedy species, this plant has many common names, including Goat's-head, Bindii, Bullhead, Burra gokharu, Bhakhdi, and Caltrop (8). Urtica dioica, often called common nettle, stinging nettle (although not all plants of this species sting) or nettle leaf, is a herbaceous perennial flowering plant in the family of Urticaceae (9). Originally native to Europe, much of temperate Asia and western North Africa, it is now found worldwide (10). Typically, in the form of creams containing antihistamines or hydrocortisone, it provides relief from nettle dermatitis. Also, its diuretic and tonic effects contribute to its wide use in painful micturition, calculus affections, and other urinary disorders (11, 12). The therapeutic value of Urtica dioica and T terrestris extracts in lowering experimental hyperoxaluria has already been reported (13–15). Besides, reports indicate that, there is a degrading bacterium in the intestine of 70-80% of healthy people which solely extracts metabolic energy from oxalate (16, 17). Further, some lactic acid bacteria (LAB) used in the dairy industry also use oxalate as energy source, potentially limiting its absorption from the intestinal lumen thereby contributing to its decreased excretion from urine (17, 18). In 2001, Campieri orally prescribed a freeze-dried preparation composed of five organisms (Lactobacillus acidophilus, L. brevis, L. plantarum, Bifidobacterium infantis, and Streptococcus thermophiles) to a few hyperoxaluria patients for 30 days. These probiotics induced a significant decrease in oxalate excretion in the patients (19, 20). In addition, dietary supplementation with probiotic has emerged as a potential strategy to increase dietary oxalate degradation (21–23). Accordingly, the purpose of our study was to evaluate the effect of simultaneous use of oxalate-degrading probiotic bacteria, Urtica dioica and T. terrestris extract in reducing urinary oxalate.

## MATERIALS AND METHODS

### Experimental animals

Male Wistar rats weighing 200-250g were used in the study. Animals were housed in a laboratory kept at 12 hours light-dark cycle, controlled room temperature (23±2°C), and relative humidity (50±10%). Also, they were exposed to 0.3% of ethylene glycol with 1% ammonium chloride in their drinking water for 3 days. Later, 0.3% ethylene glycol in drinking water was continued for 30 days (23).

### Dividing of Male Wistar rats

Male Wistar rats were divided into 4 Groups (n=6). Group I received normal diet (positive control group). Groups II (negative control group), III and IV rats received 3% ethylene glycol containing diet for 30 days. Groups III received Urtica.dioica and T. terrestris extract and Group IV rats received extracts + probiotic for 30 days.

### Preparation of hydroalcoholic extract of urtica dioica and tribulus terrestris

In this study, 60g of powder of each plant (the plants of Urtica dioica and Tribulus terrestris were used) with 300mL of ethanol 80%, were placed in a soxhlet machine for 48 hours. Then, rotary dried the extract. The experiment, the extract of the Urtica dioica was used at a concentration of 1400g/kg/body weight and from the Tribulus terrestris to 200mg/kg/body weight concentrations for each rat. Then they were dissolved in distilled water and administered twice daily by gavage (14).

### Preparation of probiotic culture

Bacterial strains used in the study were as follow: Lactobacillus acidophilus PTCC1643, Lactobacillus Delbrukii PTCC1737, Lactobacillus plantarum PTCC1745, Lactobacillus casei PTCC1608, Bifidobacterium bifidum PTCC1644, Bifidobacterium animalis subsp. lactis PTCC1736, Streptococcus salivarius subsp thermophilus PTCC1738, L. paracasei AKPL-IR (JF461540.1), and L. paracasei AKKL-IR (JF461539.1). To prepare the bacteria, 1g of dried cultures was inoculated into 100mL of MRS broth medium (Merck, Germany and placed at 37°C for 6 to 8 hours. 1mL of medium was transferred into 99mL of the new MRS broth medium and diluted to 1%. Then, it was placed at 37°C for 6 to 8 hours. The cultures transferred to the fresh medium during the week for the number of cells needed. Finally, they were kept in a refrigerator at 4°C. The probiotic cells were isolated after centrifugation at 4500rpm at 4°C for 10 minutes. Then, the separated cells were washed 2 times using the solution of 0.1% peptone (24, 25).

### Determining oxalate-degrading capability of bacterial strains

All cultures were incubated in aerobic and anaerobic conditions at 37°C for 48 hours. Base media containing ammonium oxalate was prepared to determine the growth of each strain and its dependence on oxalate, as the energy source comparing the base media lacked ammonium oxalate. Finally, all selected-strains were cultured in an ammonium oxalate plate (20mM, 40mM and 60mM) to assess their oxalate degrading capability (24, 25).

### Survival in a low pH environment and bile salts

Probiotic strains were evaluated for their resistance to low pH and bile salts. Bacterial cells were suspended in the MRS broth adjusted with 1N HCl to pH 2.5. The cells were incubated anaerobically at 37°C and their survival was measured at intervals of 0, 30, 60, 120, 180, and 240 minutes using the plate count method. Also, resistance to bile was examined using MRS agar plates supplemented with 0.5%, 1.0%, and 5.0% (w/v) bile (Oxgall; 70168 sigma). Lactobacillus species probiotic were inoculated into MRS broth and incubated at 37°C under anaerobic conditions for 24 hours. Strains were spot inoculated (10μL) onto the various concentrations of bile plates and incubated at 37°C under anaerobic conditions for 48 hours. The growth rates on porcine bile plates were compared to the growth rate on MRS agar plates and recorded (24, 25).

### Collecting urine and serum samples

On 0, 15^th^, and 30^th^ days of the study period the rats were placed in metabolic cages and 24-hour urine samples were collected in tubes containing sodium azide (0.02%) to prevent bacterial growth. The specimens were aliquoted for various tests after determining their volume and pH. Urinary oxalate, calcium, and creatinine were assayed using the commercial kit (Darman Keve Res, Lab, Isfahan, Iran) in semiautomatic photometer according to manufacturer's protocol. Each week, 1-hour urine samples were collected before the start of 24 hours urine sample collection and were examined by light microscopy to analyze CaOx-crystalluria. The rats were anesthetized with Xylasin/Ketamine by intramuscular(IM) injection (Xylasin 0.55 and Ketamine 10mg/kg Body weight) and blood was taken from orbital sinus into the centrifuge tube without anticoagulant, allowed to clot at room temperature, and centrifuged to collect serum. Sera were tested for creatinine and blood urea nitrogen (BUN).

### In vivo urinary oxalate levels using selected probiotic

Probiotics (∼10^11^ CFU) prepared in distilled water were given to the rats (intervention group) for 4 weeks (6 animals in each group). Rats were weighed weekly and urine samples were collected on weeks 0, 2, and 3, and 4 by placing the animals in metabolic cages for 24 hours.

### Analysis of histopathology and CaOx crystal in kidney

In this study, CaOx crystal present in each kidney tissue was examined by pizzolato staining methods. At first, the kidney tissue was fixed in 10% neutral buffered formalin, trimmed, processed, and set in paraffin. Sections from each kidney were stained with hematoxylin and eosin and examined under the light microscope for pathological analysis and polarized light microscope for visualizing CaOx crystal. The presence of CaOx crystal was scored on a basis of CaOx. Pathological analysis was examined with the help of the qualified pathologist.

### Statistical analysis

The statistical analysis of the antibody level between different groups was performed based on one-way and two-way ANOVA using SPSS version16 and MSTACT software. Statistical significance was set at p <0.05.

## RESULTS

**Figure 1:**
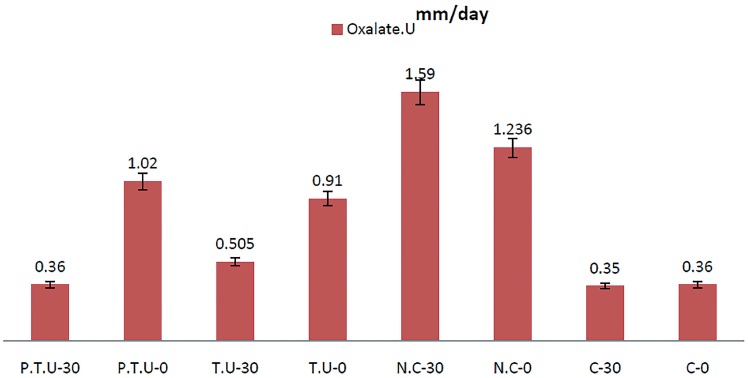
Urinary Oxalate level in all groups in 0- and −30 daysw.

Based on the results of day zero (0-day), the urine oxalate was high in all groups (except for the positive control group). But, on 30^th^ days of the study period, concentrate urinary oxalate in the negative control group was significantly higher than in the intervention and positive control groups (p <0.05), but there was no significant difference between the urinary oxalate concentrations among of the intervention and control groups. Comparison of urinary oxalate concentrations in each group during the 30 days revealed that urinary oxalate concentrations in intervention groups decreased significantly on 30^th^ day compared with the first day; in particular, in the intervention group (simultaneous use of Urtica dioica and T. terrestris extract with probiotic) the urinary oxalate concentration was normal. This suggests that probiotics are more effective than use of plant extracts, alone and without probiotic, to reducing urinary oxalate (Figure-1).

The statistical analysis of urine calcium and creatinine concentration showed a significant difference between the groups (except in the negative control group) on both days 0 (zero day) and 30 (30^th^ day). Specifically, the urinary calcium and creatinine on day 0 were high in the intervention and negative control groups and were normal in the positive control group. However, on the 30^th^ day, the urinary calcium and creatinine were still high in the negative control group, but there was a significant decrease in the intervention group (simultaneous use of Urtica dioica and T. terrestris extract with probiotic) (Tables 1 and 2).

**Table 1:** Urinary Oxalate, Calcium and Creatinine levels in Positive and Negative Control groups in 0- and −30 days.

Group		Oxalate(mm/day)	Creatinine(mg/day)	Calcium(mg/day)
*C-0	Mean	.3667	759.8333	1.7267E2
	Std. Deviation	.04131	40.42977	1.50155E1
	Minimum	.31	689.00	155.00
	Maximum	.41	800.00	189.00
C-30	Mean	.3533	715.6667	1.8417E2
	Std. Deviation	.04967	52.68649	6.24233
	Minimum	.29	677.00	177.00
	Maximum	.41	799.00	195.00
**N-0	Mean	1.2367	1412.8333	3.6567E2
	Std. Deviation	.17259	34.18430	5.35413
	Minimum	1.05	1356.00	358.00
	Maximum	1.50	1450.00	371.00
N-30	Mean	1.5967	1394.0000	4.6233E2
	Std. Deviation	.28994	16.13691	1.03473E1
	Minimum	1.08	1365.00	445.00
	Maximum	1.90	1412.00	471.00

* **C** = Positive Control;

** **N** = Negative Control

All values are expressed as Mean; Std. Deviation, Minimum, Maximum; (n=6) animals in each group.

**Table 2:** Urinary Oxalate, Calcium and Creatinine levels in Co-treatment groups in 0- and −30 days.

Group		Oxalate(mm/day)	Creatinine(mg/day)	Calcium(mg/day)
*PTU-0	Mean	1.0250	1420.3333	2.8067E2
	Std. Deviation	.06442	30.57886	1.00731E1
	Minimum	.90	1389.00	265.00
	Maximum	1.08	1459.00	291.00
PTU-30	Mean	.3650	688.1667	1.2400E2
	Std. Deviation	.06656	21.29241	6.03324
	Minimum	.55	654.00	115.00
	Maximum	.72	716.00	132.00
TU-0	Mean	.9100	1249.1667	2.8167E2
	Std. Deviation	.06841	68.86920	5.35413
	Minimum	.81	1150.00	276.00
	Maximum	.99	1329.00	289.00
TU-30	Mean	.5050	759.6667	1.7950E2
	Std. Deviation	.08735	102.49228	5.00999
	Minimum	.42	671.00	175.00
	Maximum	.63	889.00	189.00

* PTU0 = probiotic, *T Terrestris, Urtica Dioica*

All values are expressed as Mean; Std. Deviation, Minimum, Maximum; (n=6) animals in each group.

**Figure 2:**
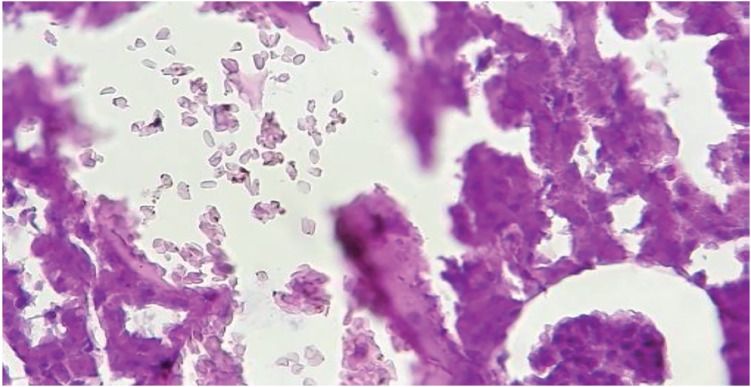
Accumulation of calcium oxalate crystals in kidney tubules in the negative group.

**Figure 3:**
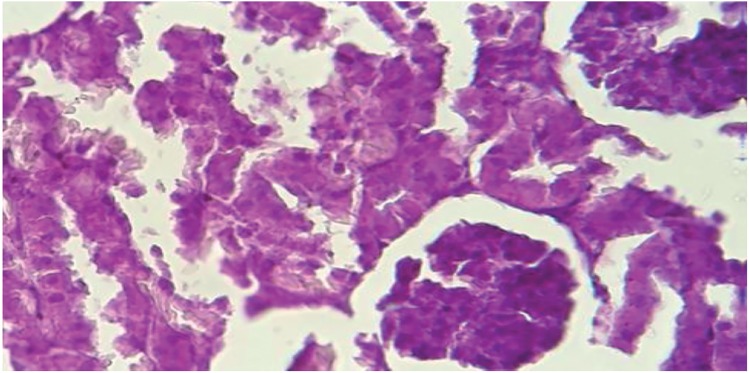
Accumulation of calcium oxalate crystals in kidney tubules in the cotreatment group (plant extracts with probiotic).

Having compared the staining results and counting the number of CaOx crystals in the kidney, in negative control groups and intervention groups, there was no accumulation of calcium oxalate crystals across the crystalline deposition in the intervention group and the positive control group (after 30 days). On the other hand, in all negative control groups, changes in pathology and physiology, including changes in the size of the kidney, changes in the color of the tissue, and an increase in calcium oxalate crystalline concentrations were observed. Therefore, the relative decline in forming crystals in the intervention group suggests a positive effect of simultaneous use of Urtica dioica and T. terrestris extract with probiotics to reduce the accumulation of CaOx crystals (Figures 2 and 3).

Also, in the serum analysis, concentrate creatinine and BUN at zero day and 30^th^ day were higher in the negative group than in other groups, and this difference was significant between the negative and intervention groups. Also, serum creatinine comparison on 30^th^ day showed reduction of creatinine and BUN on 30^th^ day compared with the first day in the intervention group (simultaneous use of Urtica dioica and T. terres- tris extract with probiotic bacteria), which was more significant. Hence, within 30 days, the level of serum creatinine and BUN in the intervention group was not significantly different with that in the positive control (Table-3). As seen in Tables 1-4, changes were observed in the level of urinary and serum parameters at the end of the treatment period (after 30 days). In the healthy control group (C-group), changes in urinary and serum parameters were not observed at the end of the period (p >0.05). But, in the negative control group (N-group) that consumed ethylene glycol daily for 30 days, at the end of the period, increased calcium oxalate crystals and renal impairment, were a significant association with ethylene glycol consumption)p <0.05(. And in Table-4, it is shown that there was a decrease in the levels of urinary oxalate and the amount of calcium oxalate crystals deposition in the two groups treated with herbal extracts alone (TU-group) and the group that consumed probiotic bacteria with plant extracts (PTU-group), there was a significant correlation between the use of probiotic with herbal extracts and reducing of urinary parameter(P <0.05). In other words, the use of herbal extracts with probiotics significantly decreased urinary oxalate levels compared to the negative control group. As shown in Tables 1-4, there was a significant difference in the level of urinary and serum parameters after the treatment period in rats (P <0.05).

**Table 3:** Serum BUN, Creatinine levels after 30days.

Creatinine(mg/dL)	BUN(mg/dL)	Groups
0.62+0.25	38.39+0.85	Positive Control
0.08	0.09	P_value_
1.82+1.05	73.19+0.13	Negative Control
0.03	0.04	P_value_
0.81+2.12	40.31+0.75	TU
0.02	0.03	P_value_
0.72+1.05	37.29+0.25	PTU
0.03	0.02	P_value_

All values are expressed as mean ± SEM; (n=6) animals in each group

**Table 4:** The change of urine oxalate, urine creatinine and the level of urinary calcium before and after the administration of plant extracts and probiotic.

The change of urinary Calcium Before/ after treatment	The change of urinary Creatinine Before/ after treatment	The change of urinary oxalate Before/ after treatment	Variables Groups
1.0217 E2	**489.49**	**0.41**	TU-Group
0.03	**0.02**	**0.04**	P value
1.566 E2	**732.17**	**0.66**	PTU-Group
0.03	**0.01**	**0.02**	P value

## DISCUSSION

Formation of urinary stones is the third most common disease in the genitourinary system, it is still considered as a chronic disease and there is no definitive treatment to prevent it (26). Up to 80% of kidney stones are predominantly composed of calcium oxalate (CaOx). Increasing urinary oxalate (hyperoxaluria) is a major risk factor for CaOx stone formation 2, 3. This formation of Calcium Oxalate stones is more due to the imbalance in the consumption of oxalate-rich foods (such as spinach, almonds, cashews, grits, beets), meat products, and the lack of expression of a number of enzymes necessary for the degradation of food oxalate, all of which reduce the risk of calcium oxalate kidney stones (2–4). Extraction of stones from different parts of the urinary tract requires advanced methods, including Extracorporeal Shock Wave Lithotripsy (ESWL), Transurethral Uretero-Lithotripsy (TUL), and Percutaneous Nephrolithotomy (PCNL), but they are very expensive (27, 28). Sometimes the accumulation of calcium or ammonium oxalate crystals in the urinary system can cause pelvic inflammation and kidney tissue, which is associated with pain and bleeding (29, 30). Also, the presence of kidney stones leads to renal diseases, including renal dysfunction, kidney tissue damage, acute or chronic renal failure, all of which can ultimately lead to kidney transplantation (31). The global prevalence of kidney stones is between 10% and 15% (32). In Iran, the prevalence of calcium oxalate stones is 61.25%, and stones with ammonium oxalate and calcium phosphate compounds claim 31.25% of cases (32, 33). Today, studies suggest a direct relationship between the recurrence of calcium oxalate stones formation and the deficiency of oxalate degrading bacteria in the gastrointestinal tract. Also, other studies, show that taking herbal extracts can help to reduce hyperoxaluria in a rat model and reduce urinary oxalate excretion in humans (34, 21).

Our study shows that feeding a mixture of probiotic bacteria with herbal extract led to a significant reduction of the excretion of oxalate in a group of rats with calcium-oxalate urolithiasis. However, an important point that supports the relevance our results is that no discrepancy was observed among those subjects regarding the impact of the experimental therapy on hyperoxaluria. We have shown, using in vitro and in vivo models, that certain probiotics offer a therapeutic strategy for reducing urinary oxalate excretion. Other studies have revealed that Lactobacillus casei and Lactobacillus plantarum can reduce the level of urinary oxalate in patients with kidney stones offering a 50% reduction (21). Similarly, Guida (2014) examined the effect of a mixture of Lactobacilli on urinary oxalate excretion in people with intestinal hyperoxaluria, with the results revealing a significant decrease in the urinary oxalate level (35). On the other hand, nowadays, with the new medical science approaches to herbs, new therapeutic ways have been proposed to improve various diseases including kidney stones (32, 15). The urinary excretion of oxalate indeed was significantly reduced by treatment with lactic acid bacteria in each of the rat groups in our study.

The treatment was associated with a mean reduction for in oxaluria, but even this change may be biologically important. In fact, only a relatively significant difference in urine supersaturation appears to exist between stone formers and non-stone formers and oxaluria rather than supersaturation has been suggested to be the most important variable for stone formation (25, 26). An additional point is that we do not exactly know what change in urine supersaturation is associated with a decrease in stone formation. It is clear fact, that even a small change in the absolute urinary excretion of oxalate could lead to substantial changes in supersaturation. Since ancient times, extracts and medicinal plants have been used as traditional treatments for urinary tract diseases such as kidney stones (15). In recent years, various plant and traditional drugs have been proposed to reduce calcium oxalate kidney stones which may be helpful in its prevention and treatment (16).

Hariprasath in 2013 stated that treatment with T. terrestris extract can prevent the rise in serum and urine levels of urinary markers such as BUN, urea, uric acid, and creatinine (14). Also, according to a Moradian study in 2017, Urtica dioica plant reduced urinary oxalate levels in rats with calcium oxalate kidney stones (36). Further, the study of Urtica dioica indicated that the plant extract at a concentration of 50 and 100mg decreased tissue inflammation.

The present study is a new approach about reducing urinary oxalate level, with concurrent use of herbal extracts with probiotic, there were interesting results. The study was conducted over a 30-day period. Before the beginning of the study, the groups were treated with ethylene glycol (to form calcium oxalate crystals in the renal tissue). Subsequently, the two main groups were examined, that included a group of rats that use herbal extracts alone (T.U--Group) and another group that use of herbal extracts with probiotic (P.T.U-Group). Initially, it was observed that the use of ethylene glycol, increased the level of urinary oxalate and the formation of calcium oxalate crystals in the kidney tissue, Which led to inflammation and renal defect, kidney malformation, and inflammation in other tissues of the rats. However, with consumption of plant extracts (without probiotic), there was a significant decrease in the level of renal inflammation and decrease in the level of urinary parameters (P <0.05). But the time to reduce renal inflammation and urinary oxalate, in this group was thirty days. While the use of herbal extracts with probiotic bacteria, reduced the inflammation and eliminated calcium oxalate crystals in the renal tissue in shorter time (20 days). The faster reduction of physiological complications and tissue inflammation can be due to the use of probiotics such as plantarium (immunosuppressive and inflammatory).

And second reason, can be due to use of two strains L. paracasei AKPL-IR (JF461540.1), L. paracasei AKKL-IR (JF461539.1), that, particularly, degrade of oxalate in vitro and in vivo, Consequently, urinary oxalate is reduced faster, which ultimately reduces the complications of the pathobiology sooner. They are submitted in NCBI site (as strains that have high power in oxalate decomposition).

Undoubtedly, this can be a new suggestion for treatment of kidney stone; however, study on the simultaneous use of herbal extracts with oxalate degrading probiotics that affect urinary oxalate excretion, needs further investigation.

## CONCLUSIONS

In this study, it was observed that at first, rats tested with kidney stones with ethylene glycol had inappropriate physiological complications, including weight loss, skin inflammation and low urine output, but at the end of the course, all of the pathogenesis rats treated with herbal extracts and probiotics appeared to be healthy and well-weighed. Importantly, rats tolerated the treatment well. Use of probiotic food supplement seems highly feasible for preventing absorptive/enteric hyperoxaluria. Future studies should also consider to evaluate an effective oxalate degrading symbiotic and plant extracts (probiotic+prebiotic+medical plant extract) and studying this combination using in vitro and in vivo studies.
